# Assessing the Effect of Treatment Duration on the Association between Anti-Diabetic Medication and Cancer Risk

**DOI:** 10.1371/journal.pone.0113162

**Published:** 2014-11-24

**Authors:** Anna But, Haining Wang, Satu Männistö, Eero Pukkala, Jari Haukka

**Affiliations:** 1 Hjelt Institute, University of Helsinki, Helsinki, Finland; 2 Department of Endocrinology and Metabolism, Peking University Third Hospital, Beijing, China; 3 Department of Chronic Disease Prevention, National Institute for Health and Welfare, Helsinki, Finland; 4 Finnish Cancer Registry, Helsinki, Finland; Baylor College of Medicine, United States of America

## Abstract

Most studies that have evaluated the association between anti-diabetic medication and cancer risk have suffered from methodological drawbacks. To avoid time-related biases, we evaluated the effect of treatment duration on the cancer risk among naive users of anti-diabetic medication as compared to non-users. In addition, we addressed the influence of common risk factors such as smoking and BMI. The study population comprised 23,394 participants of FINRISK surveys. Data on cancer and anti-diabetic medication were linked with the study cohorts. We applied Lexis tabulation to the data and analyzed split records by using Poisson regression. Changes in cancer incidence in relation to treatment duration were examined by modeling the rate ratio (RR). After a median follow-up of 9 years, 53 cancer cases among users of anti-diabetic medication and 1,028 among non-users were diagnosed. No significant difference in cancer risk between users and non-users was observed after adjustment. The RR for all medication regardless of its duration was 1.01 [95% CI 0.75–1.33], and 1.37 [0.94–1.94] for period of 1–4 years. The results were similar for metformin, sulfonylurea, and insulin. This study demonstrates that evaluation of the variation in cancer risk in relation to treatment duration is of particular importance for enhancing the accuracy of conclusions on the link between exposure to anti-diabetic medication and cancer risk.

## Introduction

Numerous epidemiological studies have demonstrated an association between diabetes and cancer risk, although the findings have not been entirely consistent [1], [2]. The underlying mechanisms are still unclear, but it is suggested that risk factors common to both disorders may explain this association to some degree. On the other hand, a causal effect of elevated glucose levels resulting from an absolute or relative deficiency of insulin as well as an effect of anti-diabetic medication (ADM) might be responsible for the potential biological link between the two diseases. It is also possible that the association between diabetes and cancer risk is affected by a combination of these factors [3].

Although there is extensive evidence for the association between ADM and cancer risk, the interpretation of results is not straightforward due to the variety of approaches to the study design and analysis used, heterogeneity of comparator populations, inadequate control for confounding, and time-related biases potentially involved [4]–[6]. The complexity of both diabetes and cancer along with the wide range of treatment options in diabetes and methodological problems have created a demanding challenge for research into the link between ADM and cancer risk. Recently, the key role of evaluation of the dosage and/or duration effect in capturing the true association between ADM and cancer risk has been emphasized [7]–[9].

In this study on FINRISK cohorts, we aimed to examine the relationship between cancer risk and ADM. In particular, our aim was to analyze the variation in cancer risk as a function of the duration of ADM and to explore the role of the common risk factors in the association of interest.

## Materials and Methods

### Study population

The study population comprised three samples from independent cross-sectional population surveys, known as the National FINRISK Study, conducted in 1997, 2002, and 2007. The main aim of the FINRISK surveys, carried out at five-year intervals since 1972, has been to assess the levels of coronary risk factors in the population of Finland [10]. For each survey, a sample was randomly drawn from the Finnish Population Information System, stratified by sex, 10-year age groups, and geographical areas according to the standardized international protocols [11]. The study was conducted according to the guidelines laid down in the Declaration of Helsinki, and the study protocol was approved by the Ethics Committee of Helsinki and Uusimaa Hospital. All study participants signed the informed consent form. The survey included a self-administered questionnaire, which was sent by mail to all selected subjects together with an invitation to a health examination. The questionnaire was completed in advance at home and was reviewed during the health examination when measurements were carried out.

The surveys were conducted in the provinces of North Karelia and Kuopio in eastern Finland, in southwestern Finland, in the province of Oulu and Lapland in Northern Finland, and in the cities of Helsinki and Vantaa (the metropolitan area). Approximately half of the total Finnish population of 5.5 million lives in the sampled regions, which incorporate both urban and rural areas. In FINRISK surveys, the participation rates have been relatively high despite the continuous decline, showing a slight regional variation and being consistently lower for men as compared to women, for which rates were 72–81% in 1997 and 69–73% in 2007 for the age group common for all surveys [10]. The surveys were carried out using both a questionnaire and health examination in all regions except Lapland in 2007, where survey was limited to the questionnaire alone. There were also those outside Lapland who participated by returning questionnaire only. In 2007, more than 65% of women took part in the survey in its entirety. Only those FINRISK participants who both returned the questionnaire and underwent the health assessment were included in the study population, which comprised 24,812 individuals. In 2007, more than 65% of women took part in the survey in its entirety.

In this study, we considered only the first primary cancer of any site except for skin cancer other than melanoma, and regarded all cancers combined as the main outcome of interest. In addition, two subgroups of cancer sites that are known to be related to either tobacco smoking or obesity were formed based on epidemiological evidence (Table S1) [12]. The follow-up time was defined for each individual as the time from entering the FINRISK study in 1997, 2002, or 2007 until December 31, 2010, the diagnosis of any cancer, or the date of death, whichever happened first. The date of entering FINRISK was determined according to the date the individual visited the study site, which took place from January to March of the survey year.

### Potential confounding variables

Based on the questionnaire responses, the individuals were classified as never, ex-, and current smokers and were divided into three groups according to their alcohol consumption. The number of drinks consumed during the week preceding examination was enquired and each drink was converted to 12 g of pure alcohol. We distinguished those not using alcohol (never or no alcohol exposure for the last 12 months), moderate users (<168 g or 14 portions per week for men, <84 g or 7 portions per week for women) and heavy users (≧168 g/week for men, ≧84 g/week for women). BMI was calculated as the weight in kilograms divided by the square of the height in meters measured at baseline. BMI was divided into four categories according to WHO recommendations [13]. Individuals with BMI less than 18.5 kg/m^2^ were categorized as underweight, 18.5–24.9 kg/m^2^ as normal weight, 25–25.9 kg/m^2^ as overweight, and 30 kg/m^2^ or more as obese. There were missing values for BMI, smoking, and alcohol consumption. In order to analyze all available data on cancer and drug exposure, for each variable with missing values we assigned an additional category denoted as “missing”.

### Register data

Incident cancers were identified by linkage with the Finnish Cancer Registry, which has collected data on all incident cancer cases in Finland since 1953 [14]. Those with a history of cancer at baseline (N = 870) were excluded from the study. FINRISK data were also linked to the register of death records of Statistics Finland (http://www.stat.fi). All individuals who had started anti-diabetic medication (ADM) after entering FINRISK and before December 31, 2010, were identified from the Prescription Register of the Finnish Social Insurance Institution [15]. This register contains information on all prescribed medicines that have been purchased since 1995. Each prescription record includes a personal identity number, the date of purchase, the ATC code of the medicine, the number of packages purchased and the disease code assigned for reimbursement approval [16]. Information on the size of the packages purchased was not available. We excluded the prevalent users of ADM (N = 548) by applying a half-year wash-out period. The final sample comprised 23,394 individuals (11,184 men and 12,210 women).

### Exposure

Although FINRISK data included information on the self-reported diagnosis of diabetes, and prescription records incorporated a disease code used for reimbursement approval, no specific information was available on either the type of diabetes or the date of the physician's diagnosis. It is most likely that the study population treated with ADM consisted of both type 1 and type 2 diabetic patients. As reliable separation of type 1 and 2 was not possible, all individuals with ADM were simply considered as diabetic patients. 

ADM aims at lowering blood glucose levels, and includes a variety of medicines working in different ways. In this study, we considered the effect of well-established medicines, which were classified into five treatment groups according to their ATC codes: metformin, sulfonylurea (including glinides), insulin and its analogues, thiazolidinedione (TZD), and others. Due to the small number of users, TZD/other medicines were only analyzed as a part of the total exposure to ADM. Oral anti-diabetic medication (OADM) was defined as anti-diabetic medicines other than insulin and its analogues.

Individuals who started ADM during the study period were considered as naive users. To avoid uncertainty in the sequence of ADM and cancer diagnoses, we regarded the cancer cases diagnosed within one month after the initiation of medication as being diagnosed in non-users. After first purchase, individuals were regarded as users, regardless of nonpersistence with medication. For each treatment group and ADM as a whole, a variable indicating the status of drug use (user, non-user) as well as a variable representing the time since initiation was specified.

### Statistical analyses

The data were processed using Lexis tabulation [17]. The individual follow-up times were cut by the start of ADM and/or by the start of a particular treatment, and split by the calendar time and time since initiation of ADM (3, 6, 12, 24, and 48 months) into intervals of short length (<0.7 years).

Based on tabulated data, a binary representation of exposure to ADM as a whole with the categories ‘no ADM’ (reference) and ‘ADM’, and treatment-specific representation with the categories ‘no ADM’ (reference), ‘other than treatment of interest’, and ‘treatment of interest’ were constructed. In addition, we defined an otherwise similar variable for each treatment but with the category ‘treatment of interest’ extended to three categories representing the duration of treatment (≤1, 1–4, >4 years).

For each potential risk factor, the cancer incidence rate ratio (RR) was assessed by applying a univariate Poisson regression model. We used an offset term equal to the natural logarithm of person-time to account for the differences in population size between subgroups. In addition, the odds ratio (OR) was evaluated using univariate logistic regression to quantify the contribution of the same risk factors to the likelihood of initiating ADM.

Poisson regression was used to examine the ADM and its specific treatments. RRs for users relative to non-users were calculated from the univariate models as well as models adjusted for gender, current age, calendar time (defined as the midpoint of each interval), BMI, and smoking. The effect of age was modeled by using a restricted cubic (natural) spline with the knots at quartiles calculated for cancer cases. For the calendar time, the use of a categorical variable with categories defined according to quintiles was found to be more suitable. The significance of each term added to the model was tested by performing an analysis of deviance with the χ^2^ test. Model selection was based on both the Akaike information criterion and comparison between the models using the deviance test. The **z**-values following a standard normal distribution were used to test against a two-sided alternative hypothesis that the model coefficients were equal to zero. The p-values corresponding to the z ratio were calculated and p<0.05 were considered statistically significant.

Graphical output for the variation in RR according to treatment or diabetes duration was provided by using restricted cubic splines. Knots for spline function were defined using quartiles calculated for cancer cases. The first knot corresponding to the start of metformin or sulfonylurea treatment was assigned to zero to allow for allocation of the effect of other ADM already being used.

All statistical analyses were performed and graphs prepared using the package Epi in R, which is free and open-source software [18].

## Results

After a median follow-up of 9 years, 1,028 cancer cases were diagnosed among 22,093 non-users and 53 among 1,301 individuals who started ADM during the study period (Table 1). Metformin was the most commonly used medicine (N = 1188), followed by sulfonylurea (N = 365) and insulin (N = 232). Cancers of the prostate, breast, female genital organs, the colorectal tract and the lung accounted for the majority of cases (Table S2). The crude incidence rate assessed for all cancer sites was higher in users of ADM, being highest in insulin users. Similar differences were seen for the cancer sites related to tobacco smoking, while only two cancer cases of sites related to obesity were found among users of ADM (Table S3). Users of ADM were older at baseline and comprised more men and obese individuals (BMI ≧30). Among users, there were more former smokers and fewer who had never smoked at baseline, and they more frequently reported not drinking alcohol. The median exposure duration was around 3 years for ADM and its treatment groups, except for sulfonylurea, for which it was almost 6 years. More than 40% of the ADM users and most of the insulin and sulfonylurea users had undergone combined therapy.

**Table 1:** Baseline and exposure characteristics among non-users and those who started ADM during the study period, 1997–2010.

	No ADM (N = 22,093)	ADM (N = 1,301)	Type of ADM		
			Metformin (N = 1,188)	Sulfonylurea (N = 365)	Insulin (N = 232)
Cancer cases, n	1,028	53	42	22	11
Median follow-up time (IQR)	8.8 (3.9–13.7)	8.9 (8.8–13.8)	8.9 (8.8–13.8)	13.6 (8.8–13.8)	13.3 (8.8–13.9)
Crude IR/1,000 PY (95% CI)	5.35 (5.02–5.69)*	9.93 (7.44–13.00)	9.27 (6.67–12.53)	10.27 (6.43–15.55)	12.46 (6.22–22.30)
Age	48.0±13.5*	61.0±11.1	61.1±10.9	62.0±10.9	60.9±12.4
Gender, male %	47.2%*	58.7%	57.8%	64.1%	68.3%
FINRISK year					
1997	6928 (31.4%)*	633 (48.5%)	568 (47.7%)	240 (66.3%)	143 (61.6%)
2002	8256 (38.6%)	472 (36.2%)	436 (36.6%)	105 (29.0%)	75 (32.3%)
2007	6906 (31.3%)	199 (15.3%)	186 (15.6%)	17 (4.7%)	14 (6.0)
BMI (kg/m^2^)					
<18.5	158 (0.7%)*	/	/	/	/
18.5–24.9	8026(36.4%)*	96(7.4%)	68 (5.7%)	28 (7.7%)	23 (9.9%)
25.0–29.9	7958 (36.0%)	458 (35.2%)	411 (34.5%)	135 (37.3%)	81 (34.9%)
> = 30	3429 (15.5%)	725 (55.8%)	696 (58.5%)	189 (52.2%)	121 (52.2%)
Missing	2522 (11.4%)	22 (1.7%)	15 (1.3%)	10 (2.8%)	7 (3.0%)
Smoking					
Never	11525 (52.2%)*	594 (45.7%)	552 (46.4%)	157 (43.4%)	102 (44.0%)
Former	4861(22.0%)	346 (26.6%)	318 (26. 9%)	102 (28.2%)	60 (25.8%)
Current	5436 (24.6%)	323 (24.8%)	287 (24.1%)	91(25.1%)	62 (26.7%)
Missing	271 (1.2%)	38 (2.9%)	31 (2.6%)	12 (3.31%)	8 (3.5%)
Alcohol consumption					
None	7820 (35.4%)*	525 (40.3%)	486 (40.9%)	159 (43.5%)	100 (43.1%)
Moderate	10375 (47.0%)	499 (38.4%)	455 38.3%)	131(34.3%)	81 (34.9%)
Heavy	3136 (14.2%)	198 (15.2%)	181 (15.2%)	42 (13.2%)	31 (13.4%)
Missing	762 (3.4%)	79 (6.1%)	66 (5.6%)	33 (9.0%)	20 (8.6%)
Median exposure duration (IQR)	/	3.2 (1.6–6.1)	3.0 (1.5–5.6)	5.6 (2.9–8.5)	3.0 (1.2–5.7)
Combined therapy^†^ (%)	/	531 (40.8%)	506 (42.6%)	320 (87.7%)	196 (84.5%)

^†^Combined therapy: treated with ADM of more than one type (insulin, metformin, sulfonylurea, TZD, others).

^*^Compared with those without ADM, P < 0.001.

Abbreviations: ADM, anti-diabetic medication; N, size of the population; n, number of incident cancer cases; IQR, inter-quartile range; IR, incidence rate; PY, person-years; CI, confidence intervals.

Aging, male gender, overweight, obesity, and smoking, both current and past, were all associated with an increased risk of both cancer and the initiation of ADM when their effect was modeled separately (Table S3). The risk of cancer as well as initiating anti-diabetic treatment was found to be lower in those with moderate use of alcohol when compared to non-drinkers, while no significant difference in cancer risk was seen between non-drinkers and heavy drinkers.

According to the Akaike information criterion and deviance test, the best fit was provided by a Poisson regression model with adjustment for age, gender, calendar time, BMI, and smoking, also including interactions between age and sex, and age and BMI. RR calculated for all cancer sites regardless of ADM duration was 1.86 [95% CI 1.39–2.42] in the unadjusted model (Table 2). After adjustment for age, gender, and calendar time (Model I), and further adjustment for BMI and smoking (Model II), no association was seen. The results were similar for metformin, sulfonylurea, and insulin analyzed separately. The crude RR of tobacco-related cancers was higher in users of ADM when compared to non-users, but after adjustment no differences were found (Table S4).

**Table 2:** Risk ratio of cancer incidence for users of ADM vs. non-users according to treatment.

	1,000 PY	Cancer cases, N	Crude IR/1,000 PY (95% CI)	Crude RR (95% CI)	*p* value	RR, Model I (95% CI)	*p* value	RR, Model II (95% CI)	*p* value
No ADM	192.21	1,028	5.35 (5.02–5.69)	1.00 (reference)		1.00 (reference)		1.00 (reference)	
Any ADM	5.34	53	9.93 (7.44–13.00)	1.86 (1.39–2.42)	<0.001	1.08 (0.81–1.42)	0.574	1.01 (0.75–1.33)	0.926
Metformin exposure									
ADM except metformin	0.81	11	13.66 (6.82–24.45)	2.55 (1.32–4.39)	0.002	1.32 (0.69–2.31)	0.367	1.28 (0.66–2.21)	0.418
Metformin	4.53	42	9.27 (6.67–12.53)	1.73 (1.25–2.32)	<0.001	1.03 (0.72–1.34)	0.829	0.96 (0.69–1.30)	0.799
Sulfonylurea exposure									
ADM except sulfonylurea	3.19	31	9.70 (6.59–13.78)	1.81 (1.24–2.54)	0.001	1.17 (0.80–1.65)	0.382	1.09 (0.74–1.55)	0.898
Sulfonylurea	2.14	22	10.27 (6.43–15.55)	1.91 (1.22–2.85)	0.002	0.99 (0.63–1.47)	0.950	0.93 (0.57–1.39)	0.718
Insulin exposure									
ADM except insulin	4.45	42	9.43 (6.79–12.75)	1.76 (1.28–2.37)	<0.001	1.02 (0.74–1.38)	0.876	0.95 (0.69–1.29)	0.771
Insulin	0.88	11	12.46 (6.22–22.30)	2.33 (1.21–4.00)	0.005	1.43(0.74–2.46)	0.242	1.36 (0.70–2.36)	0.308

Model I: Adjusted for age, gender, calendar time.

Model II: Adjusted for age, gender, calendar time, BMI, smoking status, interaction of age and gender, age and BMI.

Abbreviations: ADM, anti-diabetic medication; N, number of incident cancer cases; IR, incidence rate; PY, person-years; RR, risk ratio; CI, confidence intervals.

**Figure 1:**
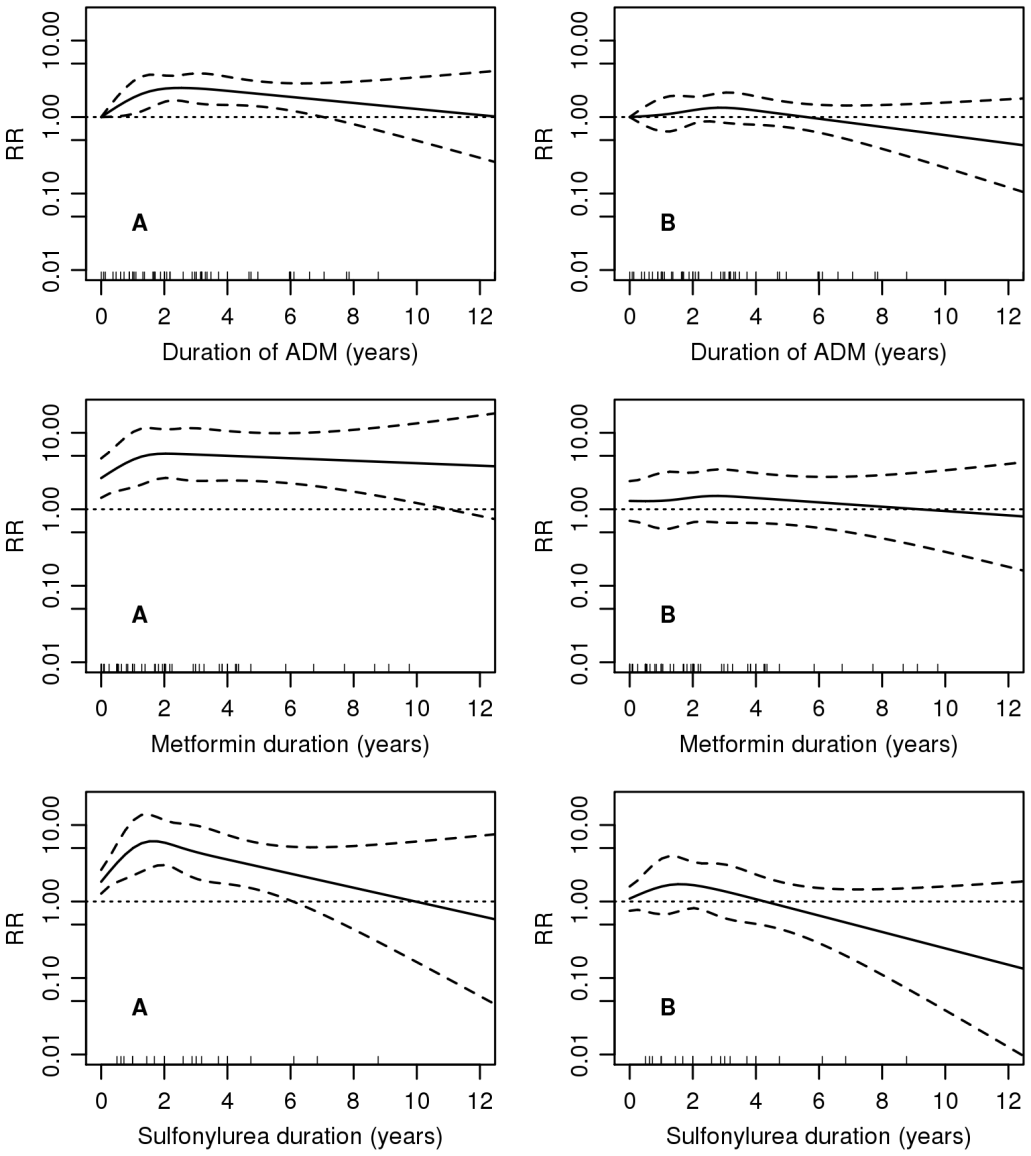
Cancer incidence rate ratio for ADM users vs. non-users. Results from spline models A - with no adjustment, B - adjusted for age, gender, calendar time, BMI, and smoking status (including interactions of age and gender; age and BMI). For metformin and sulfonylurea, allocation of RR >1 at the initiation refers to the effect of being already treated with other anti-diabetic medicines. Thick dashed lines indicate 95% CIs, thin horizontal dotted line is a reference line for no effect, tick marks along the base of plot for cancers occurred among users.

Duration analysis for any ADM yielded RRs ranging from 1.10 [95% CI 0.47–2.12] for the first year to 2.44 [95% CI 1.67–3.43] during a period of 1–4 years, and 1.58 [95% CI 0.91–2.54] for more than 4 years since the initiation, when no adjustment was performed (Table 3). Although the RR of 1.47 [95% CI 1.00–2.06] from Model I demonstrated an increased risk for the period of 1–4 years, the results from Model II did not reveal any significant difference in cancer risk between users and non-users. Similarly, comparison between metformin users, as well as sulfonylurea users and those without ADM resulted in the RRs indicating an increased risk during 1–4 years of exposure in the unadjusted model, and no differences in the risk after full adjustment. While some increase in RR during the first years after ADM initiation and a gradual decline during following years could be suggested based on the results from the unadjusted models, no noticeable variation in risk remained after adjustment for age, gender, calendar time, BMI, and smoking (Figure 1). The results for tobacco-related cancers suggested similar changes in RR in relation to exposure duration, although no significant differences in risk were detected between users and non-users after adjustment (Table S4).

**Table 3:** Variation in the cancer risk ratio in relation to exposure duration for users of ADM compared to non-users.

	1,000 PY	Cancer cases, N	Crude IR/1,000 PY (95% CI)	Crude RR (95% CI)	*p* value	RR, Model I (95% CI)	*p* value	RR, Model II (95% CI)	*p* value
No ADM	192.21	1,028	5.35 (5.02–5.69)	1.00 (reference)		1.00 (reference)		1.00 (reference)	
ADM duration									
ADM ≤1 year	1.19	7	5.86 (2.35–12.07)	1.10 (0.47–2.12)	0.810	0.68 (0.29–1.32)	0.312	0.65 (0.28–1.26)	0.250
ADM 1–4 years	2.37	31	13.07 (8.87–18.56)	2.44 (1.67–3.43)	<0.001	1.47 (1.00–2.06)	0.036	1.37 (0.94–1.94)	0.087
ADM >4 years	1.77	15	8.47 (4.74–13.98)	1.58 (0.91–2.54)	0.076	0.85 (0.49–1.37)	0.547	0.79 (0.45–1.28)	0.380
Metformin duration									
ADM except metformin	0.81	11	13.66 (6.82–24.45)	2.55 (1.32–4.39)	0.002	1.34 (0.69–2.31)	0.367	1.28 (0.66–2.21)	0.418
Metformin <1 year	1.09	5	4.59 (1.49–10.72)	0.86 (0.31–1.85)	0.734	0.53 (0.19–1.15)	0.169	0.51 (0.18–1.10)	0.131
Metfromin 1–4 years	2.10	28	13.34 (8.86–19.30)	2.50 (1.67–3.56).	0.002	1.46 (0.99–2.09)	0.030	1.41 (0.94–2.02)	0.079
Metformin >4 years	1.35	9	6.69 (3.06–12.71)	1.25 (0.60–2.26)	0.503	0.67 (0.32–1.21)	0.285	0.64 (0.31–1.17)	0.187
Sulfonylurea duration									
ADM except sulfonylurea	3.19	31	9.70 (6.59–13.78)	1.81 (1.24–2.54)	0.001	1.26 (0.86–1.78)	0.202	1.19 (0.81–1.69)	0.345
Sulfonylurea <1 year	0.35	3	8.47 (1.74–24.76)	1.58 (0.39–4.12)	0.426	0.80 (0.20–2.08)	0.702	0.77 (0.19–2.02)	0.659
Sulfonylurea 1–4 years	0.87	13	15.00 (7.98–25.66)	2.80 (1.87–5.22)	<0.001	1.46 (0.80–2.41)	0.179	1.40 (0.77–2.33)	0.226
Sulfonylurea >4 years	0.92	6	6.51 (2.38–14.17)	0.80 (0.25–1.88)	0.633	0.64 (0.25–1.30)	0.277	0.61 (0.24–1.24)	0.226
Insulin duration									
ADM except insulin	12.67	42	9.43 (6.79–12.75)	1.76 (1.28–2.37)	<0.001	1.02(0.74–1.38)	0.877	0.95 (0.69–1.29)	0.771
Insulin ≤1 year	0.20	3	14.73 (3.03–43.04)	2.75 (0.68–7.15)	0.079	1.71 (0.42–4.44)	0.355	1.64 (0.41–4.27)	0.394
Insulin 1–4 years	0.40	5	12.58 (4.04–29.13)	2.35 (0.84–5.03)	0.056	1.44 (0.51–3.10)	0.420	1.35 (0.48–2.92)	0.501
Insulin >4 years	0.28	3	10.76 (2.22–31.46)	1.99 (0.50–5.23)	0.226	1.21 (0.30–3.16)	0.738	1.18 (0.29–3.08)	0.773
Duration of OADM									
OADM only	4.45	42	9.43 (6.79–12.75)	1.76 (1.28–2.37)	<0.001	0.99 (0.72–1.33)	0.909	0.95 (0.68–1.28)	0.741
OADM ≤3y and insulin	0.30	5	16.68 (5.41–38.93)	3.12 (1.12–6.72)	0.011	2.26 (0.81–4.88)	0.054	2.28 (0.82–4.94)	0.066
OADM >3y and insulin	0.58	6	12.46 (6.22–22.30)	1.92 (0.76–3.91)	0.110	1.05 (0.42–2.14)	0.861	1.02 (0.41–2.09)	0.953

Model I: Adjusted for age, gender, calendar time.

Model II: Adjusted for age, gender, calendar time, BMI, smoking status, interaction of age and gender, age, and BMI.

Abbreviations: ADM, anti-diabetic medication; OADM, oral anti-diabetic medication; N, number of incident cancer cases; IR, incidence rate; PY, person-years; RR, risk ratio; CI, confidence intervals.

We found no elevated risk associated with the duration of insulin treatment divided into periods of ≤1, 1–4, and >4 years when compared to those without ADM (Table 3). The start of insulin treatment within 0–3 years after the initiation of OADM was associated with a higher cancer risk, RR being 3.12 [95% CI 1.12–6.72] when no adjustment was performed. However, no association was found after adjustment [RR 2.28, 95% CI 0.82–4.94]. The results did not indicate any association between the use of insulin and cancer when insulin treatment commenced more than three years after the initiation of OADM.

## Discussion

In this study on FINRISK cohorts, we analyzed the effect of ADM on cancer risk. The findings of the study do not provide evidence of either beneficial or adverse effects of anti-diabetic medication on cancer risk. One possible explanation for this can be the small number of ADM users and cancer cases among them compared to the non-users, which results in insufficient power to detect the overall association between ADM and cancer risk. Likewise, due to the limited data, the potential for heterogeneous association between different cancer types and ADM could not be addressed comprehensively, as the stratification by cancer sites was not possible. Overall, the association between ADM and cancer risk, which may exist for some cancers but not others, could not be ruled out based on the results of this study.

Primary focus of this study was to determine exposure to ADM in terms of treatment duration and to assess its effect on cancer risk. Several studies have demonstrated that time-varying representation of exposure is a meaningful component when exploring the link between diabetes or ADM and cancer risk [7], [8], [19]. Indeed, comparison of the results from analyses accounting for the duration of ADM with those from analyses ignoring the duration effect attests to the importance of considering timing.

In addition, we addressed the role of factors influencing the risk of both diabetes and cancer. We found a considerable discrepancy between diabetic and non-diabetic populations in the distribution of risk factors such as age, gender, BMI, and smoking. Moreover, we observed that these factors modify both the cancer risk and the probability of starting ADM, with patterns of risk being relatively similar. Only a limited number of observational studies have accounted for the effect of common risk factors, and there is still a need for a thorough evaluation of the impact of confounding factors [20], [21].

The changes in RRs observed as a result of the adjustment are in accordance with knowledge of the strong modifying effect of age, gender, BMI, and smoking on cancer risk. Increasing age is related to an increased risk of many diseases, including diabetes and cancer [22], [23]. Aging is also related to an increase in BMI, and thus can also affect the choice of medication [24]. Obesity is the most common co-morbidity of diabetes, and it is strongly associated with an increased risk of type 2 diabetes, as well as with higher risk of certain cancers [25]. Smoking is another lifestyle factor that is known to be related to both cancer and diabetes [26], [27]. Thus, only taking into account age, gender, and calendar time, which is the case in nationwide studies, may lead to biased results.

To draw conclusions on causality, both short-term and long-term exposure effects should be explored. In this study, the exposure of interest was specified in a manner allowing assessment of the variation in risk in relation to exposure duration. Although no significant association between the duration of ADM and cancer risk was found, the results revealed a disadvantage of analyses performed without considering the treatment duration due to the averaging of risk values over the entire period.

Some previous cohort studies have considered detection bias, reverse causality and/or indication bias as the plausible explanations for the peak in cancer risk found at the time of diabetes onset or in the early period after treatment initiation [7], [8], [19]. The development of cancer can be accompanied by an abnormal metabolic process, which is likely to be diagnosed first [9], [12], [28]. On the other hand, detection bias can be introduced due to the extensive screening usually following the initiation of ADM, which can then be associated with an increased probability of the early detection of occult cancer. This type of bias can probably be addressed by performing analysis that accounts for cancer severity at the onset or the number of physician visits [29].

We found that those who have used ADM, particularly metformin, for 1–4 years as well as those who started insulin use within 0–3 years after the initiation of oral anti-diabetic medication might be at higher risk of cancer. Reverse causality and bias in cancer detection might also be encountered at the time of initiation when starting a new treatment, especially if the initial one has not been beneficial or effective, and an additional or alternative treatment is necessary. The choice of a particular medicine as the first option and the sequence of adding new medicines to the treatment indicate the severity of diabetes and presence of comorbidities [30]. Metformin is the first-line treatment for most patients with type 2 diabetes, while insulin secretagogues and insulin are usually added on later if needed [31]. Thus, rapid progression from the initiation of OADM to the requirement for insulin treatment can be a sign of a worsening health status and can be seen as a potential risk factor for cancer. On the other hand, indication bias might be present due to the allocation of treatment, as assignment is based on strong recommendations [32].

In this study, high-quality register data from the Finnish Cancer Registry and Prescription Register allowed the effective exclusion of individuals with a cancer history at baseline and prevalent users of ADM. By using new-user design we avoided prevalent user bias, while by defining the start of follow-up similarly for both users and non-users, and by applying appropriate method of analysis we eluded immortal time bias, which arises from the misclassification of the person-time. In addition, it was possible to evaluate the variation of in cancer risk due to the technique of data analysis used. Moreover, the information on common risk factors recorded in the FINRISK survey made it possible to address potential confounding, which is one of the key biases in identifying causal effects. The participation rates in the FINRISK surveys were relatively high in the period considered by this study, making non-response bias unlikely.

One of the disadvantages of the study was the inability to distinguish between diabetes types, although only eight individuals started with insulin treatment under the age of 35 years, suggesting a minor contribution of patients with type 1 diabetes. Seemingly, also a lack of the date of diabetes onset is a subject likely to affect the results. Diabetic patients are at highest risk of cancer at the time of diagnosis of diabetes, though an increased cancer risk may even be present in the prediabetic period [7], [8], [33]. Indeed, the actual duration of exposure to elevated insulin levels cannot be reliably assessed even when precise date of diagnosis was known, as insulin resistance can predate the diagnosis of diabetes by up to 10 years [34]. In Finland, about 25% of patients diagnosed with diabetes are not included in the Prescription Register, either because of not receiving medication at all or because of inpatient care [35]. Thus, in this study some diabetic patients are likely to be included into reference population. This kind of disadvantage seems to be common to observational studies, as up to 50% of those with diabetes remain undiagnosed in European countries [36]–[38]. Other noteworthy limitations in this study were the lack of information on dosage and the analysis performed for all cancer sites combined.

Well-designed observational studies carried out using high-quality data sources and based on large samples with a long follow-up time and extensive covariate information, including the date of the onset of diabetes, treatment details, and common risk factors, would provide the greatest efficiency in exploring the relationship between ADM and cancer risk, especially if only a minor effect of ADM on the cancer risk could be suggested. Obviously, one of the key issues is the examination of cancer risk, which, if carried out according to specific sites, enables the capturing of the biological link between ADM and cancer risk. Furthermore, the detection of an association not only relies on the quality of data and the study design, but also on the approach used to represent the drug exposure. Along with the effect of ADM/diabetes duration, other relevant aspects such the dose–response relationship and drug persistence should be considered, if detailed information on medication is available. Given such complexity, it is important to use the appropriate analysis techniques and to accurately interpret the findings.

## Supporting Information

Table S1 **Number of cancer cases and crude incidence rate for the cancer sites related to tobacco smoking and obesity.**
(DOCX)

Table S2 **Number of cancer cases and crude incidence rate according to the cancer site.**
(DOCX)

Table S3 **Risk ratio (RR) of cancer incidence and odds ratio (OR) of anti-diabetic medication for the different risk factors.**
(DOCX)

Table S4 **Risk ratio of cancer related to tobacco smoking or obesity for the users of any ADM as compared to non-users.**
(DOCX)
