# Supplementary material for: Assessing the Effect of Treatment Duration on the Association between Anti-Diabetic Medication and Cancer Risk
Source: PLoS One. 2014 Nov 24;9(11):e113162. doi: 10.1371/journal.pone.0113162 (PMC4242520; doi:10.1371/journal.pone.0113162)
Supplement: Table S1 — Number of cancer cases and crude incidence rate for the cancer sites related to tobacco smoking and obesity. (DOCX) [file pone.0113162.s001.docx]

Table S1. Number of cancer cases and crude incidence rate for the cancer sites related to tobacco smoking and obesity.

| Risk factor | Number of cancer cases (IR / 1,000 PY, 95% CI) | Cancer site | ICD code |
| --- | --- | --- | --- |
| Tobacco smoking | 225 (1.14, 0.99-1.30) | Lung | C34 |
|  |  | Larynx | C 32 |
|  |  | Mouth and tongue | C01, C02, C04, C05, C06 |
|  |  | Pharynx | C11,C13,C14 |
|  |  | Esophagus | C15 |
|  |  | Pancreas | C 25 |
|  |  | Urinary bladder and renal pelvis | C65.9 C67.9 |
|  |  | Renal parenchyma | C64.9 |
| Obesity | 47 (0.24, 0.17-0.32) | Gall bladder and bile tract | C23.9 C24.9 |
|  |  | Endometrium† | C54.1 |
|  |  | Renal parenchyma† | C64.9 |
| Obesity* (females only) | 27 (0.26, 0.17-0.38) | Gall bladder and bile tract | C23.9 C24.9 |
|  |  | Endometrium^f^ | C54.1 |

† Females only

* Gender-specific incidence rate (there were in total 197.6 thousand person-years, among which 103.4 thousand were for women)

Abbreviations: IR, incidence rate; PY, person-years; CI, confidence intervals
