# Supplementary material for: Assessing the Effect of Treatment Duration on the Association between Anti-Diabetic Medication and Cancer Risk
Source: PLoS One. 2014 Nov 24;9(11):e113162. doi: 10.1371/journal.pone.0113162 (PMC4242520; doi:10.1371/journal.pone.0113162)
Supplement: Table S2 — Number of cancer cases and crude incidence rate according to the cancer site. (DOCX) [file pone.0113162.s002.docx]

Table S2. Number of cancer cases and crude incidence rate according to the cancer site.

|  | Number of cancer cases | Crude IR / 1,000 PY (95% CI) | ICD code |
| --- | --- | --- | --- |
| Prostate* | 226 | 2.40 (2.10-2.74) | C61 |
| Malignant neoplasms of the breast* | 207 | 2.00 (1.74-2.29) | C50 |
| Malignant neoplasms of the female genital organs* | 96 | 0.93 (0.75-1.13) | C51-58 |
| Malignant neoplasms of the colorectal tract | 94 | 0.48 (0.38-0.58) | C18-20 |
| Malignant neoplasms of the lung | 93 | 0.47 (0.38-0.58) | C34 |
| Malignant neoplasms of the urinary tracts | 64 | 0.32 (0.25-0.41) | C 64-68 |
| Malignant neoplasms of ill-defined, secondary, and unspecified sites | 49 | 0.25 (0.18-0.33) | C76-80 |
| Malignant neoplasms of the digestive tract, except colorectal cancers | 47 | 0.24 (0.17-0.32) | C15-17 |
| Malignant neoplasms of the central nervous system, eyes | 45 | 0.23 (0.17-0.31) | C69-72 |
| Malignant neoplasms of the liver, bile ducts, and gall bladder | 32 | 0.16 (0.11-0.23) | C22-C24 |
| Malignant neoplasms of the pancreas | 32 | 0.16 (0.11-0.23) | C 25 |
| Malignant neoplasms of the hematological and reticula-endothelial system | 27 | 0.14 (0.09-0.20) | C42 |
| Malignant neoplasms of the lip, oral cavity, and pharynx | 22 | 0.11 (0.07-0.17) | C00-14 |
| Malignant neoplasms of other respiratory organs and thoracic organs | 19 | 0.09 (0.06-0.15) | C30-33, C37- 38 |
| Malignant neoplasms of the endocrine glands | 11 | 0.06 (0.03-0.10) | C73-75 |
| Malignant neoplasms of other male genital organs* | 5 | 0.05 (0.02-0.12) | C60,  C62-63 |
| Malignant neoplasms of mesothelial and soft tissues | 8 | 0.04 (0.02-0.08) | C 45-49 |
| Malignant neoplasms of bone and articular cartilage | 4 | 0.02 (0.006-0.052) | C41 |

*Incidence rate calculated as gender-specific (there were 94.6 thousand person-years for men and 103.4 thousand for women, in total 197.6 thousand person-years)

Abbreviations: IR, incidence rate; PY, person-years; CI, confidence intervals
