# Supplementary material for: Assessing the Effect of Treatment Duration on the Association between Anti-Diabetic Medication and Cancer Risk
Source: PLoS One. 2014 Nov 24;9(11):e113162. doi: 10.1371/journal.pone.0113162 (PMC4242520; doi:10.1371/journal.pone.0113162)
Supplement: Table S3 — Risk ratio (RR) of cancer incidence and odds ratio (OR) of anti-diabetic medication for the different risk factors. (DOCX) [file pone.0113162.s003.docx]

Table S3. Risk ratio (RR) of cancer incidence and odds ratio (OR) of anti-diabetic medication for the different risk factors.

|  | 1,000 PY | Number of cancer cases | Crude IR / 1,000 PY  (95%CI) | RR* of cancer incidence  (95% CI) | p value | OR* of ADM  (95% CI) | p value |
| --- | --- | --- | --- | --- | --- | --- | --- |
| Age (years) |  |  |  |  |  |  |  |
| 25-54.9 | 116.49 | 211 | 1.81(1.57-2.07) | 1.00 (reference) |  | 1.00 (reference) |  |
| 55-64.9 | 44.30 | 340 | 7.68(6.88-8.54) | 4.24 (3.57-5.03) | <0.001 | 4.09 (3.82-4.39) | <0.001 |
| 65-69.9 | 17.40 | 201 | 11.55(10.00-13.28) | 6.38 (5.26-7.743) | <0.001 | 5.65 (5.21-6.13) | <0.001 |
| ≥70 | 19.37 | 329 | 16.98 (15.18-18.93) | 9.38 (7.90-11.16) | <0.001 | 7.08 (6.58-7.63) | <0.001 |
| Gender |  |  |  |  |  |  |  |
| Male | 94.11 | 582 | 6.18(5.69-6.71) | 1.00 (reference) |  | 1.00 (reference) |  |
| Female | 103.44 | 499 | 4.82(4.41-5.27) | 0.78 (0.69-0.88) | <0.001 | 0.61 (0.58-0.64) | <0.001 |
| FINRISK |  |  |  |  |  |  |  |
| 1997 | 96.53 | 621 | 6.43(5.93-6.96) | 1.00 (reference) |  | 1.00 (reference) |  |
| 2002 | 74.48 | 360 | 4.83(4.34-5.36) | 0.75 (0.66-0.85) | <0.001 | 0.67 (0.64-0.71) | <0.001 |
| 2007 | 26.54 | 100 | 3.77(3.06-4.59) | 0.59 (0.47-0.72) | <0.001 | 0.47 (0.43-0.52) | <0.001 |
| BMI (kg/m^2^) |  |  |  |  |  |  |  |
| <18.5 | 1.44 | 7 | 4.85(1.95-10.00) | 1.08 (0.46-2.11) | 0.842 |  |  |
| 18.5-24.9 | 73.22 | 329 | 4.49(4.02-5.01) | 1.00 (reference) |  | 1.00 (reference) |  |
| 25.0-29.9 | 74.14 | 478 | 6.45(5.88-7.05) | 1.43 (1.25-1.65) | <0.001 | 5.24 (4.71-5.84) | <0.001 |
| ≥30 | 35.31 | 241 | 6.82(5.99-7.74) | 1.52 (1.29-1.79) | 0.003 | 20.27 (18.28-22.54) | <0.001 |
| Missing | 13.43 | 26 | 1.94(1.26-2.84) | 0.43 (0.28-0.63) | <0.001 | 1.44 (1.16-1.78) | <0.001 |
| Smoking |  |  |  |  |  |  |  |
| Never | 103.44 | 465 | 4.50(4.10-4.93) | 1.00 (reference) |  | 1.00 (reference) |  |
| Former | 42.70 | 303 | 7.10(6.32-7.95) | 1.58 (1.36-1.82) | <0.001 | 1.39 (1.31-1.48) | <0.001 |
| Current | 48.95 | 281 | 5.74(5.09-6.46) | 1.28 (1.10-1.48) | 0.001 | 1.12 (1.05-1.19) | <0.001 |
| Missing | 2.47 | 32 | 12.96 (8.85-18.30) | 2.88 (1.97-4.05) | <0.001 | 2.94 (2.52-3.41) | <0.001 |
| Alcohol consumption |  |  |  |  |  |  |  |
| Non-user | 67.99 | 395 | 5.81(5.25-6.41) | 1.00 (reference) |  | 1.00 (reference) |  |
| Moderate | 93.43 | 474 | 5.07 (4.62-5.56) | 0.87 (0.76-0.99) | 0.047 | 0.72 (0.63-0.82) | <0.001 |
| Heavy | 27.47 | 138 | 5.02(4.22-5.94) | 0.86 (0.71-1.05) | 0.141 | 0.79 (0.73-0.87) | 0.418 |
| Missing | 8.66 | 74 | 8.55(6.71-10.74) | 1.47 (1.14-1.87) | 0.002 | 1.36 (1.21-1.52) | 0.002 |

* Evaluated using univariate model

Abbreviations: RR, rate ratio; OR, odds ratio; IR, incidence rate; PY, person years; CI, confidence intervals
