# Supplementary material for: Assessing the Effect of Treatment Duration on the Association between Anti-Diabetic Medication and Cancer Risk
Source: PLoS One. 2014 Nov 24;9(11):e113162. doi: 10.1371/journal.pone.0113162 (PMC4242520; doi:10.1371/journal.pone.0113162)
Supplement: Table S4 — Risk ratio of cancer related to tobacco smoking or obesity for the users of any ADM as compared to non-users. (DOCX) [file pone.0113162.s004.docx]

Table S4. Risk ratio of cancer related to tobacco smoking or obesity for the users of any ADM as compared to non-users.

|  | 1,000 PY | Cancer cases, N | Crude IR /1000 PY  (95% CI) | Crude RR (95% CI) | *p* value | RR, Model I (95% CI) | *p* value | RR, Model II  (95% CI) | *p* value |
| --- | --- | --- | --- | --- | --- | --- | --- | --- | --- |
| No ADM † | 192.21 | 210 | 1.09 (0.95-1.25) | 1.00 (reference) |  | 1.00 (reference) |  | 1.00 (reference) |  |
| Any ADM† | 5.34 | 15 | 2.81 (1.57-4.64) | 2.57 (1.46-4.19) | <0.001 | 1.23 (0.70-2.02) | 0.440 | 1.29 (0.72-2.14) | 0.362 |
| ≤1 year † | 1.20 | 1 | 0.84 (0.02-4.66) | 0.77 (0.04-3.40) | 0.790 | 0.40 (0.02-1.77) | 0.359 | 0.43 (0.02-1.90) | 0.395 |
| 1-4 years† | 2.37 | 9 | 3.79 (1.73-7.20) | 3.47 (1.65-6.37) | <0.001 | 1.74 (0.82-3.20) | 0.107 | 1.81 (0.85-3.37) | 0.089 |
| >4 years † | 1.77 | 5 | 2.82 (0.91-6.60) | 2.59 (0.92-5.63) | 0.035 | 1.12 (0.40-2.46) | 0.809 | 1.16 (0.41-2.59) | 0.749 |
|  |  |  |  |  |  |  |  |  |  |
| No ADM* | 192.21 | 45 | 0.23 (0.17- 0.31) | 1.00 (reference) |  |  |  |  |  |
| Any ADM * | 5.34 | 2 | 0.37 (0.05-1.35) | 1.60 (0.26-5.17) | 0.515 |  |  |  |  |

† Cancers related to tobacco smoking

* Cancers related to obesity

Model I: Adjusted for age, gender, calendar time

Model II: Adjusted for age, gender, calendar time, BMI, smoking status, interaction of age and gender, age, and BMI

Abbreviations: ADM, anti-diabetic medication; N, number of cancer cases; IR, incidence rate; RR, risk ratio; PY, person-years; CI, confidence intervals
